# [Retracted] Upregulation of microRNA‑375 increases the cisplatin‑sensitivity of human gastric cancer cells by regulating ERBB2

**DOI:** 10.3892/etm.2025.12799

**Published:** 2025-01-13

**Authors:** Ning Zhou, Yanli Qu, Chunlei Xu, Yong Tang

Exp Ther Med 11:625–630, 2016; DOI: 10.3892/etm.2015.2920

Following the publication of the above paper, a concerned reader drew to our attention apparent anomalies associated with the western blot data featured in Fig. 6; essentially, it appeared as if overlapping sections of gel slices had been included in the figure, where subsequently they were taken to erroneously represent the results of differently performed experiments.

After having investigated this matter independently, the Editor of *Experimental and Therapeutic Medicine* has upheld the claims made by the interested reader, and a decision has been taken that this paper should be retracted from the Journal owing to the anomalous presentations of the western blots in Fig. 6. The authors were asked for an explanation to account for these concerns, but the Editorial Office did not receive a reply. The Editor regrets any inconvenience caused to the readership of the Journal.

